# Biological and Chemical Removal of Primary Cilia Affects Mechanical Activation of Chondrogenesis Markers in Chondroprogenitors and Hypertrophic Chondrocytes

**DOI:** 10.3390/ijms17020188

**Published:** 2016-02-04

**Authors:** Matthew E. Deren, Xu Yang, Yingjie Guan, Qian Chen

**Affiliations:** 1 Cell and Molecular Biology Laboratory, Department of Orthopaedics, Alpert Medical School of Brown University/Rhode Island Hospital, 1 Hoppin Street, Suite 402, Providence, RI 02903, USA; matthew_deren@brown.edu (M.E.D.); yingjie_guan@brown.edu (Y.G.); 2 Department of Orthopaedics, Affiliated Hospital of Medical College of Qingdao University, Qingdao 266003, China; 3 Bone and Joint Research Center, the First Affiliated Hospital, Frontier Institute of Science and Technology, Xi’an JiaoTong University, Xi’an, 710054, China

**Keywords:** primary cilia, mechanotransduction, chondrocytes

## Abstract

Chondroprogenitors and hypertrophic chondrocytes, which are the first and last stages of the chondrocyte differentiation process, respectively, are sensitive to mechanical signals. We hypothesize that the mechanical sensitivity of these cells depends on the cell surface primary cilia. To test this hypothesis, we removed the primary cilia by biological means with transfection with intraflagellar transport protein 88 (IFT88) siRNA or by chemical means with chloral hydrate treatment. Transfection of IFT88 siRNA significantly reduced the percentage of ciliated cells in both chondroprogenitor ATDC5 cells as well as primary hypertrophic chondrocytes. Cyclic loading (1 Hz, 10% matrix deformation) of ATDC5 cells in three-dimensional (3D) culture stimulates the mRNA levels of chondrogenesis marker Type II collagen (Col II), hypertrophic chondrocyte marker Type X collagen (Col X), and a molecular regulator of chondrogenesis and chondrocyte hypertrophy bone morphogenetic protein 2 (BMP-2). The reduction of ciliated chondroprogenitors abolishes mechanical stimulation of Col II, Col X, and BMP-2. In contrast, cyclic loading stimulates Col X mRNA levels in hypertrophic chondrocytes, but not those of Col II and BMP-2. Both biological and chemical reduction of ciliated hypertrophic chondrocytes reduced but failed to abolish mechanical stimulation of Col X mRNA levels. Thus, primary cilia play a major role in mechanical stimulation of chondrogenesis and chondrocyte hypertrophy in chondroprogenitor cells and at least a partial role in hypertrophic chondrocytes.

## 1. Introduction

During endochondral ossification, a chondroprogenitor cell undergoes differentiation to a proliferative chondrocyte, which correlates to the synthesis of chondrogenic markers such as Type II collagen (Col II). This is followed by chondrocyte hypertrophy with the synthesis of Type X collagen (Col X) before bone formation. Skeletal formation in the developing body as well as skeletal repair in the adult relies on differentiation of cartilage [1]. The regulation of these processes is affected by stress, including mechanical stress within the cartilage, which modulates chondrocyte function through a molecular mechanism which is still largely unknown.

The primary cilium is a single extension from the apical surface of vertebrae cells that is not actively motile [2,3]. They are a microtubule-based appendage with a 9 + 0 axoneme lacking the central microtubule pair that imparts active motility [4,5,6]. Similar to the basal body, these organelles project into the extracellular matrix and are covered with a specialized plasma membrane. Long felt to be a vestigial organelle, the primary ciliium is now believed to be multifunctional antenna detecting alterations in the extracellular environment [6,7,8]. The function of this versatile organelle depends upon its structural integrity, and defects in the primary cilia have been associated with polycystic kidney disease, obesity, cancer, arthritis, and osteoporosis [8,9,10,11].

The role of the primary cilia’s interaction with signaling molecules has been further classified in recent years. The primary cilium has also been identified as center for regulating complex signaling pathways including Hedgehog and Wingless [12,13,14]. In the brain, somatostatin receptor 3 (SST_3_) and 5-hydroxytryptamine-6 (5HT6) serotonin receptors are found on primary cilia [15]. Smoothened, an essential transmembrane protein for the Hedgehog (Hh) pathway in skeletal development localizes to the membrane of primary cilia [14,16,17]. A study of primary cilia in bone cells demonstrated they deflect during dynamic fluid flow in a manner of mechanosensation independent of calcium intake, implicating them in both osteogenic and bone resorptive processes [18]. Other studies identified the function of primary cilia as flow sensors in renal tubule epithelial cells [2]. Disruption of primary cilia in growth plate chondrocytes leads to reduced bone length; disorganized growth plates; disrupted Indian Hedgehog signaling and endochondral bone formation; accelerated chondrocyte hypertrophy; and reduced chondrocyte proliferation [13,14,15,19,20,21].

Protein synthesis does not occur in primary cilia, so maintenance of these organelles requires intraflagellar transport, the shuttling of essential proteins via the microtubules from base to tip. Anterograde transport is mediated by intraflagellar transport protein 88 (IFT88), also known as Polaris, and disruption of this protein results in loss of primary cilia [19,21]. Kinesin-like protein 3a (Kif3a) is involved in retrograde intraflagellar transport, and its phenotype has been well-studied [22,23]. In knockout organisms lacking primary cilia, deletion of Kif3A reduces loading-induced bone formation [22,23].

One shortfall of previous experimental designs is the examination of cells under fluid flow using a monolayer of cells, which does not accurately represent the three-dimensional (3D) environment of chondrocytes *in vivo*. Studies using a 3D culture sponge allow for cyclic mechanical loading of chondrocytes, showing that increased local strain results in increased expression of Col II and Col X, markers for proliferative and hypertrophic chondrocyte activity, respectively [24]. Treatment of cells with chloral hydrate is an effective chemical method to remove primary cilia [25]. RNA interference is a biological method to knockdown IFT88 and removes primary cilia from cells [3,19].

In this study, we look to examine if primary cilia transduce mechanical forces into biological signals in chondroprogenitor cells and chondrocytes by comparing control chondrocytes to those treated with chloral hydrate or IFT88 knockdown while stimulating the cells in a mechanically active 3D culture sponge. We intend to examine the efficiency of removing primary cilia by immunohistochemistry and Western blot as well as expression of previously studied mechanoresponsive genes in both chondroprogenitor cells and chondrocytes.

## 2. Results

### 2.1. Disrupting Primary Cilia Structure Inhibits Cyclic Loading-Induced Mechanosensitive Genes in ATDC5 Chondroprogenitor Cells

**Figure 1:**
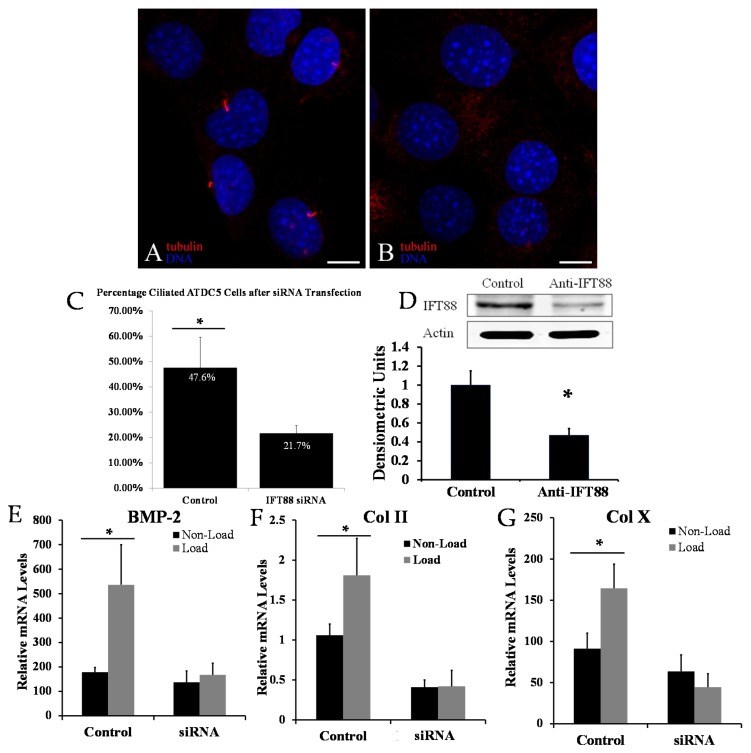
Confocal microscope image showing a field of ATDC5 mouse chondroprogenitor cells transfected with scrambled control (**A**) or intraflagellar transport protein 88 (IFT88) siRNA (**B**). Primary cilia are extending from the cell surface of the control-group cells (**A**) but not present in the IFT88 siRNA cells (**B**); acetylated α-tubulin is stained red; DNA is stained blue with DAPI (scale bars: 10 µm). IFT88 siRNA transfection decreased the number of ciliated cells by immunocytochemistry from 47.6% in controls to 21.7% (**C**); Western blot demonstrates effective knockdown of IFT88 by transient transfection. Quantitation values of IFT88 protein levels normalized to actin are presented (**D**); Significant differences in relative Type II collagen (Col II) mRNA (**E**); Type X collagen (Col X) mRNA (**F**); and bone morphogenetic protein 2 (BMP-2) mRNA (**G**) between loaded and non-loaded conditions in control transfected cells were not significant in IFT88 transfected cells. Values normalized to 18S rRNA. Statistically significant values are represented by *.

To study the effect of primary cilia in mechanical regulation of chondroprogenitor cells, we knocked down IFT88 by transfection of ATDC5 cells with IFT88 siRNA. Immunohistochemical staining against acetylated-α-tubulin was performed to identify a long, smoothly curved ciliary structure on cell surface (Figure 1A). The percentage of ciliated chondroprogenitor cells was significantly reduced in the IFT88 siRNA transfected group (21.7% ± 3%) in comparison to the control scrambled siRNA transfected group (47.6% ± 12%) (Figure 1C). A successful knockdown of IFT88 was demonstrated by decreased levels of IFT88 protein in experimental *versus* control groups by Western blot (Figure 1D).

Cyclic mechanical loading of 3D cultured ATDC5 cells significantly increased Col II, Col X and BMP-2 mRNA levels in comparison to non-loaded cells (Figure 1E–G). Interestingly, the up-regulation of these mechanosensitive genes was abolished in loaded ATDC5 cells transfected with IFT88 siRNA (Figure 1E–G). These data suggest cyclic loading promotes the differentiation of chondroprogenitor cells, and the primary cilium was required for this process.

### 2.2. Biological Reduction of the Percentage of Ciliated Chondrocytes Decreased but Did Not Abolish Cyclic Loading Stimulation of Chondrocyte Hypertrophy

**Figure 2:**
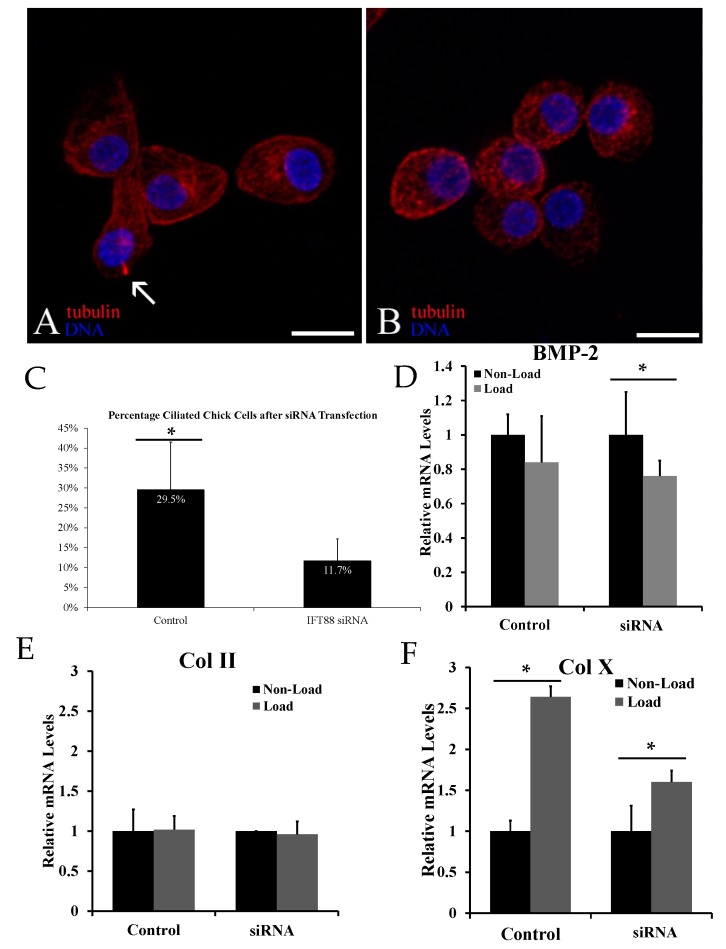
Confocal microscope image showing a field of chick primary chondrocytes transfected with scrambled control (**A**) or intraflagellar transport protein 88 (IFT88 siRNA) (**B**). Primary cilia are extending from the cell surface of the control-group cells, identified by the arrow above (**A**) but absent in the IFT88 siRNA cells (**B**); acetylated α-tubulin is stained red; DNA is stained blue with DAPI (scale bars: 10 μm). IFT88 siRNA transfection decreased the number of ciliated cells by immunocytochemistry from 29.5% in controls to 11.7% (**C**); A significant difference in relative Type X collagen (Col X) mRNA levels was present between loaded and non-loaded cells transfected with scrambled control (**D**); This statistically significant difference was reduced but still present after IFT88 siRNA transfection. There was no statistically significant difference in Type II collagen (Col II) relative mRNA levels (**E**) or bone morphogenetic protein 2 (BMP-2) relative mRNA levels (**F**). Values normalized to 18S rRNA. Statistically significant values are represented by *.

To determine whether primary cilia are also required for mechanical stimulation of chondrocyte differentiation in primary hypertrophic chondrocytes, immunohistochemistry was performed using anti-acetylated α-tubulin after transfection with IFT88 siRNA. The number of ciliated hypertrophic chondrocytes was significantly reduced in IFT88 siRNA transfected group (11.7% ± 5.5%) in comparison to control siRNA transfected group (29.5% ± 12.0%) (Figure 2C).

While cyclic loading significantly increased the mRNA levels of hypertrophic marker Col X, it failed to increase those of Col II and BMP-2, which are synthesized by pre-hypertrophic chondrocytes (Figure 2D–F). Reduction of the percentage of ciliated chondrocytes decreased but did not eliminate mechanical stimulation of Col X (Figure 2D). Biological removal of the primary cilia had no effect on the mRNA levels of Col II and BMP-2 under loading and non-loading conditions (Figure 2E,F).

### 2.3. Chemical Removal of Primary Cilia Inhibits Cyclic Loading-Induced Type X Collagen (Col X) mRNA in Hypertrophic Chondrocytes

**Figure 3:**
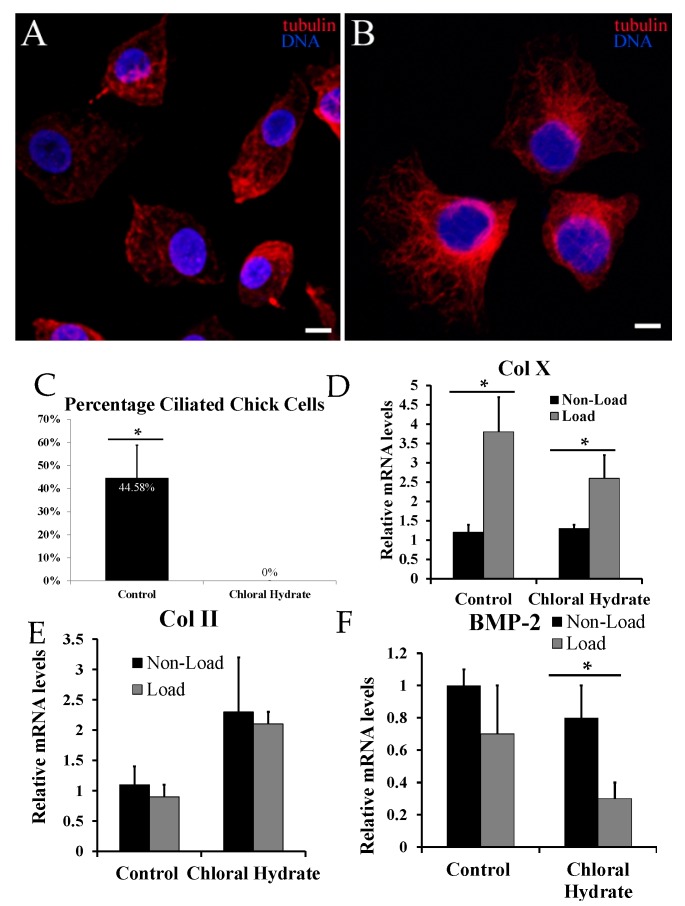
Confocal microscope image showing a field of chick primary chondrocytes treated with control (**A**) or chloral hydrate-containing culture medium (**B**). Primary cilia are red structures extending from the cell surface of the control-group cells (**A**) but absent in the chloral hydrate treated cells (**B**); acetylated α-tubulin is stained red; DNA is stained blue with DAPI (scale bars: 10 μm). Immunocytochemistry analysis indicated that treatment with chloral hydrate decreased the number of ciliated cells by from 44.6% in controls to 0% (**C**); A statistically significant difference in relative Type X collagen (Col X) mRNA was present between loaded and non-loaded cells treated with control (**D**); This statistically significant difference was still present but again smaller in those treated with chloral hydrate. There was no statistically significant difference in relative Type II collagen (Col II) mRNA levels (**E**); There was a statistically significant decrease in relative bone morphogenetic protein 2 (BMP-2) mRNA levels in loaded *versus* non-loaded chloral hydrate-treated cells which was not present in controls (**F**). Values normalized to 18S rRNA. Statistically significant values are represented by *.

Since the transfection of IFT88 siRNA reduced but did not completely eliminate all primary cilia from chondrocytes due to the transfection efficiency, we also chemically removed the primary cilia from the cell surface with chloral hydrate treatment. Immunocytochemical analysis with anti-acetylated-α-tubulin demonstrated disruption of the cytoskeleton and total abrogation of primary cilia in chloral hydrate-treated chondrocytes (Figure 3A–C).

Under non-loading conditions, chloral hydrate treatment did not affect the Col X mRNA level significantly (Figure 3D). Thus, chloral hydrate by itself did not affect the Col X mRNA level. However, chloral hydrate treatment increased the Col II mRNA level and reduced the BMP-2 mRNA level under non-loading conditions (Figure 3E,F). Under loading conditions, the Col X mRNA level in control chondrocytes increased 3.2 fold, while that in chloral hydrate treated cells only increased two fold (Figure 3D). Thus, chemical removal of primary cilia reduced but did not eliminate the mechanical stimulation of Col X mRNA. Under chloral hydrate treatment, there was no statistically significant difference in the Col II mRNA levels between loading and non-loading conditions (Figure 3E), while loading further reduced BMP-2 mRNA levels in hypertrophic chondrocytes.

## 3. Discussion

In this study, primary cilia were successfully removed from chondroprogenitor cells and primary chondrocytes by biological means with IFT88 siRNA transfection and by chemical means with chloral hydrate treatment, as indicated by immunocytochemistry and Western blot analyses. The biological method has few side effects as IFT88 siRNA transfection does not affect Col II, Col X or BMP-2 mRNA levels in chondroprogenitors or primary chondrocytes.

The incidence of primary cilia in adult articular chondrocytes, when analyzed by serial section of TEM (transmission electron microscopy) *in situ*, has been documented to approach one per cell [26]. By comparison, cells in tissue culture generally have a lower incidence of primary cilia [27]. Because the mother centriole, which typically forms one of the mitotic spindle poles, comprises the ciliary basal body, primary cilia are resorbed prior to mitosis but reassembled and present throughout the majority of interphase. Thus, actively dividing cells are expected to have a ciliary incidence of less than one per cell. In our study, primary cilia were identified based on acetylated α-tubulin by immunostaining as a projection from the cell surface. It provides a relatively easy and rapid way to assess ciliary incidence. However, it is possible that if the primary cilium was not pointing parallel to the slide surface, it may have not been detected on confocal microscopy as the primary cilium measures approximately 0.2 μm and 1–5 μm in size [28,29]. In addition, previous work by Malone *et al.* in osteocytes and osteoblasts demonstrated that 60%–62% of cells were ciliated, while this number decreased to 47.8%–58% after transfection with control siRNA. Therefore, 47% of control ciliated chondrocytes in our study is near the expected range based on previously published literature [3].

The removal of primary cilia from the cell surface by siRNA is not complete due in part to the limitation of transfection efficiency. Since the transient transfection is not 100% efficient, IFT88 cannot be expected to be knocked down and the primary cilia to be removed in all of the cells in culture. Treatment with chloral hydrate completely removes primary cilia by disassembly of the cytoskeleton in chondrocytes, but it is a non-specific method causing more disruption to the cellular architecture than transfection with IFT88 siRNA. Although chloral hydrate treatment did not affect Col X basal level mRNA expression, it altered those of Col II and BMP-2. There is no evidence to suggest that chloral hydrate in this concentration causes apoptosis of chondrocytes, and it has been used in protocols to remove the primary cilia from both canine renal epithelial cells and murine osteoblasts and osteocytes [2,3].

Removal of primary cilia from chondroprogenitor cells completely abrogated mechanical induction of chondrogenesis marker Col II, hypertrophic marker Col X, and a key regulator of chondrogenesis and hypertrophy BMP-2. This suggests that primary cilia are required for mechanical activation of chondrogenesis and hypertrophy of chondroprogenitor cells. In contrast, the role of primary cilia in mechanical stimulation of differentiated hypertrophic chondrocytes appears to be more limited, as complete removal of primary cilia reduced but did not eliminate the increase of Col X mRNA in response to mechanical loading.

Previous studies of the primary cilium have demonstrated its role in mechanical stimulation of the extracellular environment. One study in renal epithelial cells demonstrated that primary cilia were involved in the sensation of extracellular fluid flow in a two-dimensional model [3]. Likewise, the removal of primary cilia from renal epithelial cells demonstrated a decreased flow-induced calcium signaling response within the cell [2]. The results of this experiment in chondroprogenitor cells and primary chondrocytes are consistent with previous studies demonstrating the role of primary cilia in mechanotransduction.

One study described the potential role of primary cilia in the alignment of cells in the physis of bones possibly due to maintenance of cellular polarity [30]. Cells in the resting zone were found to be non-polarized, but as chondrocytes became polarized in the hypertrophic and proliferative zones of the physis, the primary cilia became positioned parallel to the long axis of the bone. Primary cilia are oriented away from the articular surface in articular cartilage [31,32]. Our study also suggests that the primary cilia of chondroprogenitor cells in the resting zone may play an important role in regulating chondrogenesis and chondrocyte differentiation in response to the mechanical environment, while those of more differentiated hypertrophic chondrocytes may play a more limited role in that regard. This observation also supports previous theories linking the mechanical environment to the initiation of endochondral ossification [1]. Primary cilia may play an important role in the chondrocyte differentiation process by chondroprogenitors in addition to its role in mesenchymal stem cells, osteoblasts, and osteocytes [33,34].

The data from both indicate that primary cilia of chondrogenic cells play a role in regulating chondrogenic and hypertrophic gene expression in response to changes in the mechanical environment. Further investigation is necessary to determine if primary cilia on the surface of chondrogenic cells are required for mechanical stimulation of endochondral ossification *in vivo*. 

## 4. Materials and Methods

### 4.1. Cell Culture and Mechanical Stimulation

Primary chick embryonic chondrocytes were isolated from the cephalic part of 17-day embryonic chick sternal cartilage and cultured in F12 medium supplemented with 10% fetal bovine serum (FBS) (Life Technology, Grand Island, NY, USA) and 1% antibiotic. The cells were seeded into a three-dimensional organotypic chondrocyte culture as previously described [35]. One million cells were applied to 2 × 2 × 0.25 centimeter^3^ (cm^3^) gelfoam sponges (Upjohn, Kalamazoo, MI, USA) presoaked with Hanks’ balanced salt solution (HBSS) (Life Technology). The 3D chondrocytes were then treated with or without 4 mM chloral hydrate for 72 h. The medium was then changed, and the cells were mechanically loaded for 24 h in fresh F12 medium with 10% cyclic load applied by the computer-controlled BioStretch system (ICCT Technologies, Markham, ON, Canada). Non-loaded sponges seeded with cells were kept at the same culture condition without cyclic loading and used as the control. At the indicated mechanical loading duration, sponges were washed thoroughly with HBSS, cut into small pieces, and digested in 0.03% (*w*/*v*) collagenase in HBSS at 37 °C for 10 min. Chondrocytes were collected by centrifugation for RNA or protein preparations.

ATDC5 mouse chondroprogenitor cells were cultured in DMEM/F12 medium (Life Technology) supplemented with 10% fetal bovine serum, 10 μg/mL human transferrin, 3 × 10^−8^ M sodium selenite, 100 U/mL penicillin, and 0.1 mg/mL streptomycin and allowed to proliferate. Upon reaching confluence, the cells were then trypsinized and seeded into 3D organotypic chondrocyte culture in the same medium plus 25 µg/mL ascorbic acid. After overnight incubation, sponges were loaded with an intermittent pattern (5% elongation, 1 Hz) for 48 h with a BioStretch device (ICCT Technologies).

### 4.2. Chemical Abrogation of Primary Cilia

For removing primary cilia, the chick chondrocytes were treated for 72 h with 4 mM chloral hydrate (Spectrum Laboratory Products, New Brunswick, NJ, USA) in F12 medium and then placed in fresh medium for another 24 h before fixation, both steps at 37 °C. Controls were incubated in F12 medium without added chloral hydrate but an equal volume of Hanks Buffered Saline Solution (HBSS).

### 4.3. Transient Transfection

Cells were transfected using the Lipofectamine 2000 system (Invitrogen, Carlsbad, CA, USA) with a 24-bp custom small interfering RNA (siRNA) targeting IFT88 (5′-CCAGAAACAGATGAGGACGACCTTT-3′) and All-Stars Negative siRNA Fluorescein control siRNA (Qiagen, Valencia, CA, USA) per manufacturer’s instructions. This target sequence is conserved between both chicken and mouse genomes. Transfected cells were cultured overnight following transfection then seeded to collagen sponges and followed by cyclic loading at the indicated time points. Transfection of ATDC5 chondroprogenitor cells with control siRNA resulted in a transfection efficiency of 62% based on fluorescein detection, while transfection of chick primary chondrocytes resulted in a transfection efficiency of approximately 52.7% using the fluorescein-labeled control for detection.

### 4.4. Immunohistochemistry

The cells were fixed in paraformaldehyde and incubated with a primary antibody of anti-acetylated α-tubulin (1:500, Sigma, St. Louis, MO, USA) overnight at 4 °C. The secondary antibody used was tetramethyl rhodamine isothiocyanate (TRITC)-conjugated donkey anti-mouse IgG (1:200, Jackson ImmunoResearch, West Grove, PA, USA) incubated at room temperature for 2 h, and the cells were then stained and mounted with VectaShield containing DAPI (Vector Laboratories, Burlingame, CA, USA). Negative controls were incubated in PBS without the primary antibody. All images were obtained using a Nikon Eclipse TE2000-E confocal microscope and edited in Adobe Photoshop (Adobe, San Jose, CA, USA).

### 4.5. Western Blotting

Total proteins extracted from cells were collected by radioimmunoprecipitation assay (RIPA) lysis buffer supplemented with proteinase inhibitors (Cell Signaling Technology, Beverly, MA, USA). Equal amounts of protein lysates were separated by sodium dodecyl sulfatepolyacrylamide gel electrophoresis (SDS-PAGE) and transferred to nitrocellulose membrane for immunoblotting with IFT88 antibodies (ProteinTech, Chicago, IL, USA). Infrared fluorescence labeled secondary antibody was detected with an Odyssey fluorescence scanner (LI-COR Biosciences, Lincoln, NE, USA). Quantification of Western blot data was performed using software in the Odyssey Infrared Imaging system.

### 4.6. Real Time Reverse Transcription Polymerase Chain Reaction (RT-PCR)

Chicken chondrocyte sponges were collected after 24 h of cyclic loading. ATDC5 cells were collected at 48 h of cyclic loading. Total RNA was isolated using the RNAqueous-4PCR kit (Ambion, Austin, TX, USA). One microgram of total RNA from mechanically loaded and non-loaded samples was used for each reverse transcriptase reaction with the iScript cDNA Synthesis kit (Bio-Rad, Hercules, CA, USA). Quantitative real time PCR was performed on the CFX96 Touch™ Real-Time PCR Detection System (Bio-Rad Laboratories, Hercules, CA, USA) using the QuantiTect SYBR green PCR kit (Qiagen, Valencia, CA, USA) per the manufacturer’s instructions. 18S rRNA was amplified at the same time as an internal control. Primer sequences used for detecting the mRNA level of chicken type X collagen α1 (Col X), type II collagen α1 (Col II), and calculations of relative transcript abundance were described previously [29]. The following sequence-specific primers were synthesized: 5′-TGGTGGAGCAGCAAGAGCAA-3′ and 5′-CAGTGGACAGTAGACGGAGGAAA-3′ for mouse collagen II; 5′-CTGCTGCTAATGTTCTTGAC-3′ and 5′-ACTGGAATCCCTTTACTCTTT-3′ for mouse Col X; and 5′-CCCGGCGCTTCTTCTTCAATT-3′ and 5′-CTGGGGTGACGTCGAAGCTCTC-3′ for mouse BMP2. Results are from three replicates from three independent experiments. The presence of a single specific PCR product was verified by melting curve analysis and confirmed on an agarose gel.

### 4.7. Statistical Analysis

Three independent experiments were performed with the results expressed as the mean ± SD. Normal distribution was confirmed using the Shapiro–Wilk test. Two tailed Student’s *t*-test and analysis of variance (ANOVA) with *post-hoc* tests were used for pairwise and multiple comparisons, respectively. Significance was accepted at the 0.05 level of probability (*p* < 0.05).
